# Case Report: Successful anti-TNF antibody therapy in steroid-dependent segmental colitis associated with diverticulosis and concomitant ulcerative colitis

**DOI:** 10.3389/fimmu.2026.1764305

**Published:** 2026-04-15

**Authors:** Fotios Fousekis, Jürgen Siebler, Timo Rath, Arndt Hartmann, Markus F. Neurath, Michael Vieth, Raja Atreya

**Affiliations:** 1 First Department of Medicine, University Hospital Erlangen, Friedrich-Alexander-Universität Erlangen-Nürnberg, Deutsches Zentrum Immuntherapie (DZI), Erlangen, Germany; 2 Institute of Pathology, University Hospital Erlangen, Friedrich-Alexander-Universität Erlangen-Nürnberg, Erlangen, Germany; 3 Institute of Pathology, Klinikum Bayreuth, Friedrich-Alexander-Universität Erlangen-Nürnberg, Bayreuth, Germany

**Keywords:** adalimumab, diverticulosis, segmental colitis associated with diverticulosis, TNF, ulcerative colitis

## Abstract

Segmental colitis associated with diverticulosis (SCAD) is a rare inflammatory condition limited to colonic segments affected by diverticulosis. Its coexistence with ulcerative colitis (UC) is extremely rare and poses a diagnostic and therapeutic challenge. We report the case of a 56-year-old woman with a history of sigmoidectomy for recurrent sigmoid diverticulitis, who subsequently developed SCAD in the residual diverticula adjacent to the anastomosis, along with concomitant left-sided UC. Despite treatment with mesalazine and vedolizumab and rectal healing of UC, SCAD was therapy-refractory. The use of the anti-TNF antibody adalimumab, intensified to 40 mg weekly, resulted in complete clinical, endoscopic, and histologic remission of both SCAD and UC. At five years of follow-up to date, the patient remains in sustained, steroid-free remission without adverse events. This case highlights the possible coexistence of SCAD and UC and suggests adalimumab as a promising and sustained treatment option in refractory SCAD, even in the setting of coexisting UC.

## Introduction

Segmental colitis associated with diverticulosis (SCAD) is an inflammatory condition affecting the mucosa of the colon in areas where diverticulosis is present, mainly in the sigmoid and descending colon. The absence of involvement in the proximal colon and rectum is a notable feature that differentiates SCAD from other inflammatory conditions. Prevalence of SCAD ranges from 2% to 11% of patients suffering from diverticulosis. Given its close association with diverticular disease, most studies have reported an average age of diagnosis in the sixties (1, 2). SCAD may cause rectal fresh or altered bleeding, chronic diarrhea, cramping abdominal pain, with up to one-third of patients showing multiple symptoms at diagnosis. Systemic features like fever, and weight loss are rare (3). SCAD shares more similarities with inflammatory bowel diseases (IBD) than with diverticular disease. Although the underlying mechanisms of SCAD remain uncertain, its pathogenesis is considered multifactorial with contributors including increased mucosal permeability, localized ischemia and dysbiosis (3).

SCAD can mimic UC both clinically and histologically. Histologically, both entities may demonstrate chronic inflammatory infiltrates in the lamina propria, neutrophilic cryptitis, crypt abscesses, basal plasmacytosis, lymphoid aggregates, crypt architectural distortion and Paneth cell metaplasia, which can complicate differentiation. However, many of these features tend to be less frequent or less pronounced in SCAD. For example, crypt abscesses have been reported in approximately 20% of SCAD cases compared with 45% in UC, while crypt architectural distortion is observed in about 7% versus 25%, respectively (4). In addition, the inflammation in SCAD is segmental and confined to areas with diverticula, while UC usually involves the rectum and may proximally extend to other colonic segments. Compared with IBD, SCAD has a more benign outcome, with a lower rate of complications (5). Coexistence of the two entities is uncommon but has been documented in the literature and careful assessment is essential for accurate diagnosis and management (6).

Here, we present the case of a patient with coexisting SCAD and UC, who was successfully treated with the anti-TNF antibody adalimumab, achieving both endoscopic and histological remission for both disease entities.

## Case presentation

A 56-year-old Caucasian female with a history of recurrent episodes of acute sigmoid diverticulitis underwent elective sigmoidectomy eight years ago. The immediate postoperative course was uneventful, and she remained asymptomatic for seven months. Subsequently, she developed recurrent left lower quadrant pain, intermittent tenesmus, and increased stool frequency with episodes of diarrhea and rectal bleeding. There was no occurrence of systemic symptoms such as fever, weight loss, or other extraintestinal manifestations. Physical examination revealed mild tenderness over the left lower quadrant without peritoneal signs. Laboratory tests showed normal white blood cell count and C-reactive protein, while fecal calprotectin was elevated (207 μg/g). Stool cultures were obtained to exclude infectious causes and were negative.

**Figure 1:**
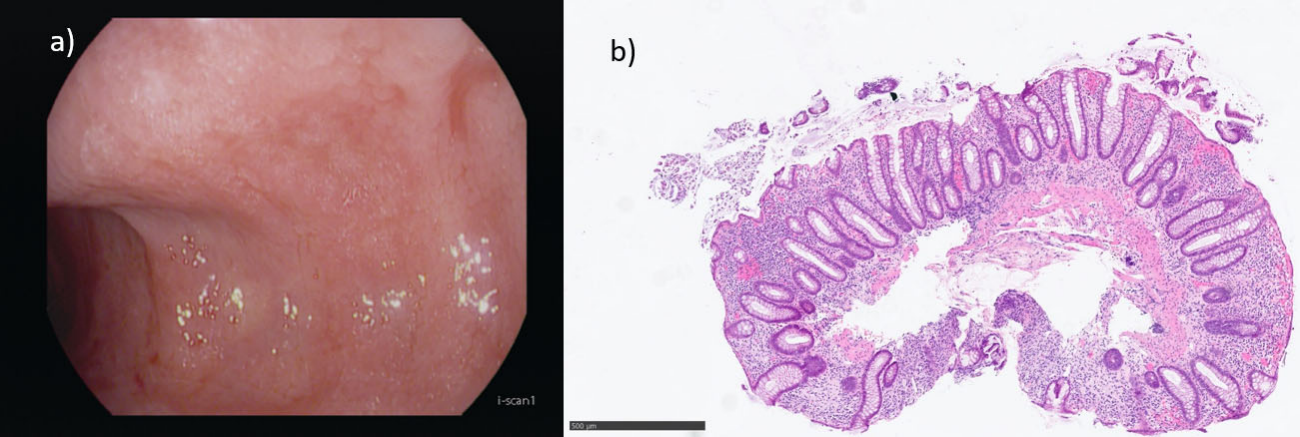
**(a)** Endoscopic image from the descending colon demonstrating colitis with continuous inflammation, erythema, friability, loss of vascular pattern, and superficial erosions. The endoscopic findings are consistent with ulcerative colitis (Mayo endoscopy score II). **(b)** Histopathological findings from rectal biopsy, consistent with ulcerative colitis, demonstrating altered crypt architecture with shortened crypts, reduced number of goblet cells, slightly elevated cell count in the lamina propria (lymphocytes and plasm cells), few eosinophils and focal Paneth cell metaplasia, with no granuloma formation.

Six months after the initiation of symptoms ileocolonoscopy was performed, revealing proctosigmoiditis with reduced vascular pattern, erythema, friability, superficial erosion from the rectum to the proximal descending colon (MAYO score II) (**Figure 1**). The remaining colon and terminal ileum appeared endoscopically with no pathological changes. Mild mucosal edema was observed at the anastomotic site, where diverticular openings were visible. Biopsies were taken from inflamed areas. Histopathological examination demonstrated features of chronic active colitis consistent with UC, including cryptitis, crypt architectural distortion, an elevated cell count within in the lamina propria, leucopedesis into the surface epithelial, reactive changes and absence of granulomas (**Figure 1**). *In-situ* hybridization against cytomegalovirus (CMV) was performed and was negative. A diagnosis of left-sided (distal) UC was established based on clinical, endoscopic and histologic findings, and treatment consisting of oral mesalazine 3.6 g/day in combination with topical budesonide 4 g once daily and oral budesonide MMX 9 mg/d was initiated for induction of remission. After three months of therapy, she achieved a partial clinical response, with reduction of stool frequency and tenesmus but with persistence of left lower quadrant pain.

**Figure 2:**
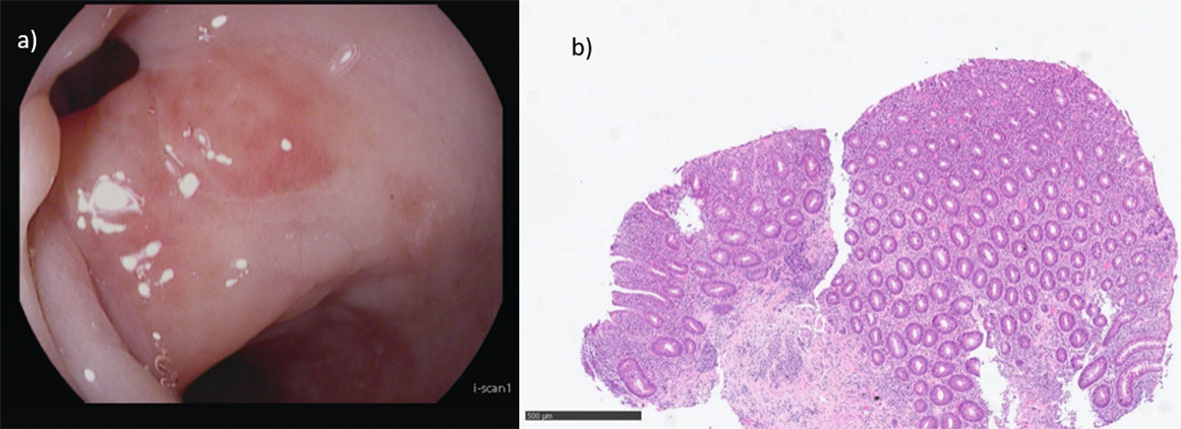
**(a)** Endoscopic image from the region surrounding the anastomosis showing residual diverticula with inflammation around the diverticular orifice, reflected by mucosal erythema and edema. **(b)** Biopsy near diverticula showing features consistent with segmental colitis associated with diverticulosis (SCAD): preserved crypt architecture, flattened and degenerated surface epithelium, increased cellularity within the lamina propria with lymphocytes and plasma cells, very few eosinophilic granulocytes and neutrophils, and a reduced number of goblet cells.

Follow-up endoscopy at that time revealed improvement of the rectal inflammation, with endoscopic mucosal healing of the previously inflamed areas and reduced disease activity in the distal colon (MAYO score I). Although rectal healing may have been influenced by the use of topical therapy, persistent inflammation was noted in the region surrounding the anastomosis, where residual diverticula were identified with edematous and erythematous mucosa, while the rectum remained spared (**Figure 2**). Histology from the colon, adjacent to diverticula, revealed preserved crypt architecture with flattened surface epithelium, mild chronic inflammation of the lamina propria, and a reduced number of goblet cells, findings consistent with reactive changes in SCAD and less typical of UC (**Figure 2**). Due to the persistence of symptoms, as well as the endoscopic and histologic findings, systemic corticosteroid therapy was initiated (prednisolone 50 mg/day). Although the patient achieved clinical remission, she was steroid-dependent, with symptomatic relapse upon dose reduction, requiring repeated courses of corticosteroids to maintain remission.

Because of the steroid-dependent course, three months later it was decided to initiate biologic therapy with vedolizumab, an anti-integrin α4β7 agent primarily targeting UC, with the expectation that it might also contribute to the remission of SCAD. Following biological screening, the patient received the standard dose of vedolizumab for 5 months, during which corticosteroids were tapered. The patient achieved a partial clinical remission with reduced stool frequency; however, she continued to report intermittent left lower quadrant pain and mild rectal bleeding. A repeat sigmoidoscopy four months after vedolizumab initiation showed ongoing inflammation confined to the area near the anastomosis, where diverticula were present, with rectal sparing, an endoscopic pattern typical of SCAD, while there were no signs of active UC.

Given the persistent activity and incomplete response to vedolizumab, a switch to an anti–TNF agent was decided due to persistent clinical symptoms (three soft bowel movements per day, intermittent left lower quadrant pain and mild rectal bleeding) and ongoing endoscopic inflammation. Adalimumab was initiated with the standard induction regimen (160 mg at week 0, 80 mg at week 2), followed by maintenance dosing of 40 mg every two weeks, leading initially to a marked clinical improvement. However, three months later, she experienced a recurrence of rectal bleeding and abdominal discomfort. Local steroid therapy with budesonide foam was added for 4 weeks, and the adalimumab dosing interval was shortened to 40 mg weekly. Reevaluation of the patient at the outpatient clinic after four weeks demonstrated a good clinical response and subsequently clinical remission.

**Figure 3:**
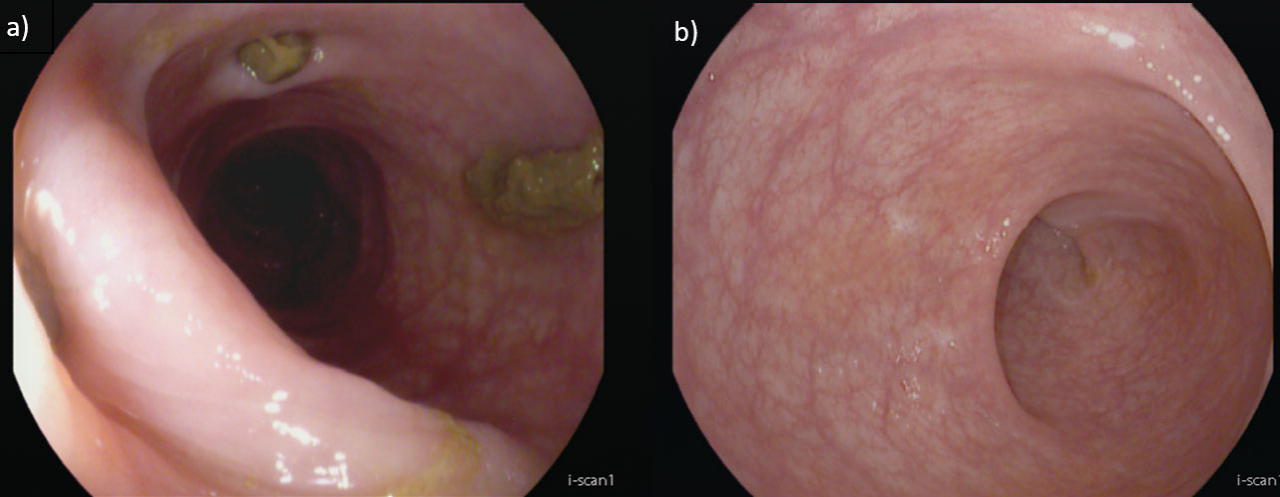
**(a)** Follow-up endoscopic images after 7 months of adalimumab weekly monotherapy showing resolution of inflammation in the diverticular segment and **(b)** complete mucosal healing in the rectum (Mayo endoscopic score 0).

**Figure 4:**
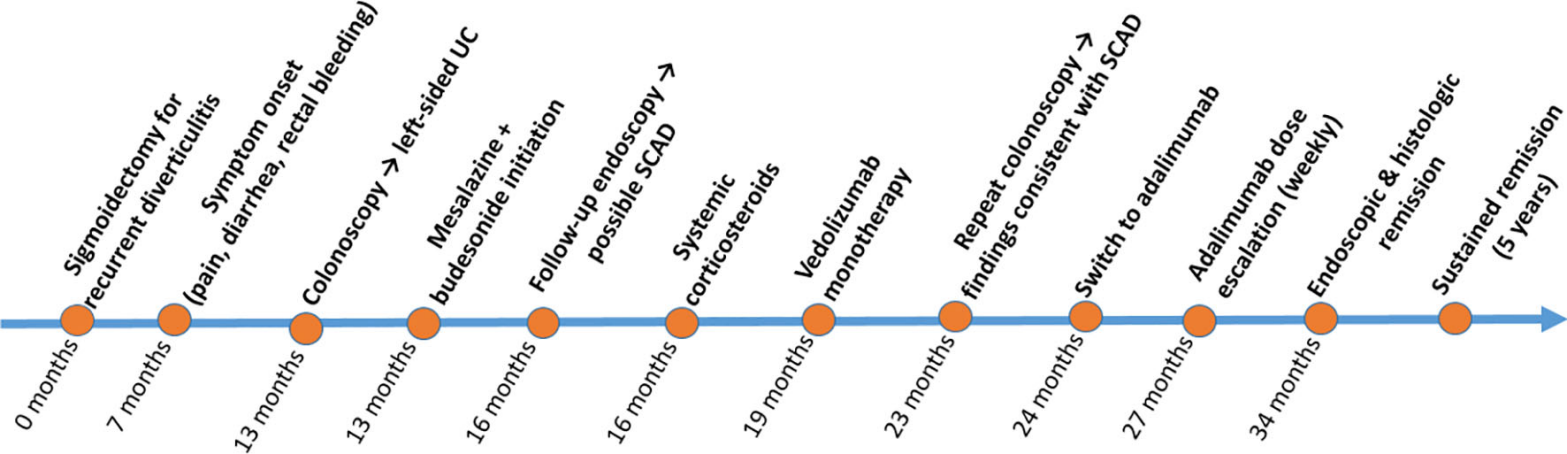
Timeline of the patient’s clinical course showing symptom onset, diagnostic evaluation, treatment interventions, and long-term clinical outcome.

Follow-up endoscopy after 7 months, during which the patient was receiving adalimumab monotherapy weekly, revealed mucosal healing in the rectosigmoid and a resolution of inflammation in the diverticular segment and the rectum appeared endoscopically normal (MAYO score 0) (**Figure 3**). Histological evaluation demonstrated mild chronic inactive colitis without active cryptitis (UC remission phase). The patient continued regular follow-up at the outpatient clinic every 3–6 months and underwent endoscopic reassessment every 1–2 years. The patient has since remained in sustained steroid-free clinical, endoscopic and histologic remission at the latest follow-up visit without adverse events, five years after therapy escalation to adalimumab 40 mg weekly (**Figure 4**). During follow-up, the patient was regularly monitored for potential anti-TNF–related adverse effects, including infectious complications, allergic reactions, and laboratory abnormalities (complete blood count and liver function tests). No adverse events or other clinical events potentially related to adalimumab therapy were reported.

## Discussion

Regarding SCAD diagnosis, laboratory and imaging studies are of limited specificity and are mainly useful for excluding other causes such as malignancy and infection. Therefore, endoscopy remains the cornerstone of SCAD diagnosis, with clear communication of clinical suspicion to the pathologist. In our case, the diagnosis of SCAD was particularly challenging due to the coexistence of left-sided UC. However, the persistence of inflammation confined to the diverticular segment after improvement of rectal disease suggested the coexistence of SCAD, highlighting the diagnostic challenge when both entities overlap. Endoscopically, SCAD may be classified into four main inflammatory patterns. The mildest form, “crescentic fold disease”, is characterized by localized hyperemia and swelling of the mucosal folds with preservation of the diverticular orifices, as observed in our case. The “mild-to-moderate UC-like” pattern resembles UC, with erythema, edema, loss of vascular pattern, and superficial erosions. The “Crohn’s-like” variant is characterized by scattered aphthous ulcers within otherwise normal mucosa. Finally, the “severe UC-like” form presents with diffuse ulceration, friability, and marked mucosal edema (2). The histological spectrum of SCAD ranges from preserved crypt architecture with acute and chronic inflammatory cell infiltrates limited to the crypt epithelium, to chronic inflammatory changes in the lamina propria, including basal plasmacytosis, goblet cell depletion, crypt architectural distortion, cryptitis, crypt abscesses, and crypt hemorrhage (2, 7).

Development of UC after diagnosis of SCAD is rare but documented and a small subset of patients with SCAD may later develop UC, characterized by extension of inflammation beyond the diverticular segment, including involvement of the rectum and other colonic segments. Based on a review including eight well-documented patients, UC typically developed within a mean interval of approximately 11 months after SCAD diagnosis. In addition, five of the eight patients who developed UC progressed to aggressive disease necessitating colectomy (8). Furthermore, a recent series identified five patients who progressed from SCAD to UC after a median of 63 weeks, all showing distal extension of inflammation with new-onset rectal involvement. Most required escalation to biologic therapy, and one underwent colectomy (9). In our case, endoscopic assessment at our center revealed active inflammation within the segment containing residual diverticula as well as in the rectum, complicating the diagnostic distinction. The persistence of peridiverticular inflammation after vedolizumab therapy and endoscopic healing of the rectum was a key finding that supported the coexistence of both entities. Moreover, the preserved crypt architecture observed in biopsies obtained near the diverticula is a feature more consistent with SCAD than with UC.

Optimal management of SCAD has not yet been established and currently relies on evidence from case series and treatment approaches used in IBD. First-line treatment of SCAD usually consists of antibiotics, salicylates, or a combination of both complemented by a high-fiber diet. Contrary to IBD, corticosteroids and other immunosuppressants are rarely used (10). In a recent cohort of 44 patients with SCAD, the most frequent subtype was the crescentic fold disease (N = 23), followed by the mild-to-moderate UC-like form (N = 10), the Crohn’s-like variant (N = 8), and the severe UC-like subtype (N = 3). During a three-year follow-up period, all patients received mesalazine therapy. Overall, 27 patients (61.4%) achieved complete remission, whereas none of those with the severe UC-like form attained full recovery. Six patients needed steroids and one patient received adalimumab to control symptoms (11). Aside from this study by Tursi et al. (11, 12), the administration of anti-TNF agents for isolated SCAD has been documented only once previously, in a case successfully managed with infliximab (13).

TNF appears to be a mediator of the inflammatory process in SCAD. Overexpression of TNF has been demonstrated in the colonic mucosa of patients with SCAD and has been associated with high histological severity of disease and neutrophilic cell account (14). Furthermore, high mucosal TNF levels have been correlated with more severe endoscopic patterns of SCAD (15). In addition, mucosal TNF levels in SCAD have been found to be significantly higher than in irritable bowel disease and comparable to those in UC and Crohn’s disease (16). Based on these clinical scarce data and the evidence supporting the role of TNF in the pathogenesis of SCAD, and given the well-established efficacy of adalimumab in UC (17), we decided to initiate this treatment. In our patient, treatment was further escalated to adalimumab weekly, possibly due to a more pronounced inflammatory burden resulting from the coexistence of UC.

The presented case emphasizes that persistent inflammation within diverticular segment in patients with UC should not be overlooked, as both entities may coexist. It also highlights the therapeutic potential of anti–TNF agents in the management of SCAD, even when coexisting with ulcerative colitis. Adalimumab achieved sustained clinical, endoscopic, and histological remission in steroid dependent SCAD after failure of vedolizumab. Despite limited evidence, this case supports anti–TNF therapy in refractory SCAD cases and underscores the need for further investigation.

## Data Availability

The raw data supporting the conclusions of this article will be made available by the authors, without undue reservation.
